# Analysis of Labial and Lingual Strength among Healthy Chinese Adults in Taiwan

**DOI:** 10.3390/ijerph17217904

**Published:** 2020-10-28

**Authors:** Shang-Jung Wu, Chun-Chieh Wang, Feng-Yu Lin, Kai-Yu Tseng, Yueh-Juen Hwu

**Affiliations:** 1 Department of Nursing, Taichung Veterans General Hospital Puli Branch, Nantou 54552, Taiwan; ernr1191@gmail.com; 2 Department of Public Health, China Medical University, Taichung 40447, Taiwan; 3 Department of Nursing, Central Taiwan University of Science and Technology, Taichung 40601, Taiwan; 107179@ctust.edu.tw; 4 Department of Internal Medicine, Taichung Veterans General Hospital Puli Branch, Nantou 54552, Taiwan; proteinmad@yahoo.com.tw; 5 Department of Eldercare, Central Taiwan University of Science and Technology, Taichung 40601, Taiwan; 6 General Education Center in the Overseas Chinese University, Taichung 40721, Taiwan; fyl@seed.net.tw; 7 College of Nursing, Central Taiwan University of Science and Technology, Taichung 40601, Taiwan

**Keywords:** tongue strength, labial strength, endurance, presbyphagia

## Abstract

This study collected 11 parameters regarding the labial and lingual strength for maximum isometric and swallowing tasks among 150 healthy Chinese adults in Taiwan. Measurements were performed using the Iowa Oral Performance Instrument (IOPI). All of the labial and lingual strength parameters were measured three times. The maximal value of three trials represents the pressure of every parameter. The overall mean (±standard deviation) and maximum isometric pressures of the lips, anterior tongue, and posterior tongue were 24.81 ± 5.64, 55.95 ± 14.13, and 53.23 ± 12.24 kPa, respectively. The mean value of posterior tongue strength was less than that of the anterior tongue by approximately 5%. The percentages of maximum isometric tongue pressure during the swallowing of saliva and water were 85% and 80% for the anterior tongue and 90% and 81% for the posterior tongue, respectively. The average endurances for the anterior tongue and posterior tongue were 13.86 ± 7.08 and 10.06 ± 5.40 s, respectively. The maximum isometric pressures were greater than both the saliva and water swallowing pressures, and the saliva swallowing pressures were greater than the water swallowing pressures. A value of 33 kPa in maximum isometric pressure could serve as a demarcation of weak tongue strength for healthy Chinese adults. As for the repeated trials of labial and lingual strength, there were no statistically significant differences for any of the pressures obtained from the 11 labial and lingual strength parameters. The normative data can be used for the objective assessment of labial and lingual strength in healthy Chinese adults.

## 1. Introduction

There is a three-stage sequential model of the process of normal swallowing, which is divided into oral, pharyngeal, and esophageal stages according to the location of the bolus. The orbicularis oris muscle is located in the lower part of the face; it is responsible for lip closure and prevents food from spilling out of the mouth during swallowing. Lip strength is measured based on the pressure generated by the orbicularis oris muscle. Decreased lip strength causes difficulty in lip closure, resulting in food leakage and a reduction in intraoral pressure that leads to swallowing impairment [1].

Regarding the function of swallowing, tongue muscles are responsible for bolus retention in the oral cavity and bolus transport from the oral cavity to the pharynx. The maximal tongue pressure exerted on the hard palate may offer a quantitative parameter for evaluating tongue motor biomechanics during swallowing [2]. The exact and objective evaluation of tongue pressure is thus important for the monitoring of swallowing and oropharyngeal movements [3].

Adams et al. [4] conducted a systematic review and meta-analysis of measurements of tongue strength and found 38 studies that addressed this purpose. Among them, 12 studies reported tongue strength data for health adults. Nine studies illustrated the values of anterior tongue strength in the healthy adult population. Three studies measured tongue strength in both the anterior and posterior position. The maximum isometric pressure (MIP) of the tongue, including the anterior and posterior regions, has a range of 43–78 kPa in healthy adults. The maximum anterior tongue strength was observed to decrease with increasing age in nine studies. Two age groups were considered (below 60 years and above 60 years) in conducting the meta-analysis. The results indicated that the maximum tongue strength of the younger adults was 10–15 kPa higher than that of the oldest adults. There were some inconsistencies with the finding that tongue strength is reduced with increasing age. Clark and Solomon [5] reported that significant main effects of age were identified for both of the anterior and posterior tongue pressure. Although both of the means for the anterior and posterior tongue pressures in the middle-age group were greater than the means for the young-age and old-age group, pairwise comparisons revealed no differences from each other. The value of 40 kPa can be used as a cut-off value to demarcate a weak or strong tongue strength for healthy older adults [6,7].

The tongue plays an important role in both speech and swallowing; however, the strength requirements for the two functions are markedly different [8]. Typically, adults use ≤20% and 40–60% of the maximum tongue strength for speech production and swallowing, respectively [9,10]. The lingual-palatal swallowing pressures generated during swallowing tasks are termed the maximum swallowing pressure (MSP). The swallowing pressure reserve is the difference between the MIP and the MSP. The MIP has been shown to decrease with age, whereas age does not appear to have a significant effect on the MSP [9,11,12].

Tongue muscle endurance is defined as the length of time one can maintain a submaximal contraction [5,13]. Healthy adults can maintain half of their maximum tongue strength for at least 25–35 s for both tongue regions [14]. A value ≤ 10 s is viewed as a negative indicator for the maintenance of adequate tongue function [4,13].

Dysphagia problems increase gradually with age and are frequently undetected or untreated [15]. The reported incidence of dysphagia in community-dwelling adults is 22.6% (mean age: 48.1 years). Of adults with dysphagia, 46% had not consulted a physician regarding their symptoms [16]. Swallowing problems can occur even in healthy order adults, which is called presbyphagia and is associated with age-related decrease in tongue muscle strength [17,18]. In Taiwan, there are four hundred thousand individuals suffering from dysphagia [19]. The availability of normative data is a prerequisite when seeking to determine the impact of lingual strength on observed swallowing disorders [20].

Some participants in a previous study reported feeling orofacial muscle soreness immediately after their tongue strength measurement, and it is described in the Iowa Oral Performance Instrument (IOPI) user manual that a participant who is to perform the tongue strength measurement may experience “throat soreness” due to normal muscle fatigue [21,22]. After the establishment of the reference data for tongue strength in healthy Chinese adults, the tongue strength measurement will be applied in a wide range of clinical practice—for example, with frail patients, individuals with physical disability, or older adults. Whether this research population can withstand the muscle fatigue during tongue strength measurement in three consecutive trials motivated us to explore the relationship between the three trials.

Therefore, the present study aimed to provide data from Chinese individuals in Taiwan by measuring the tongue endurance, the pressure exerted by the lips and tongue on the oral cavity during the maximum isometric contraction and during saliva and water swallowing. The difference in three trials in labial and tongue strength under the conditions of maximum isometric pressure, saliva and water swallowing, and tongue endurance were also investigated.

## 2. Materials and Methods

### 2.1. Participants

Participants were recruited from the general public. A total of 150 healthy adults participated in this study. Adults aged >20 years with normal lips, teeth, tongue, palate, and chewing were included. Those with a history of orthodontic treatment or temporomandibular disorders, a history of major surgery of the head or neck, oral disease, or a history of neurologic impairment were excluded. Written informed consent was provided by all the participants for inclusion in this study. Approval for data collection was obtained from the Research Ethics Committee of Jen-Ai Hospital (no. 107-47).

### 2.2. Lingual and Labial Strength Evaluation

The Iowa Oral Performance Instrument (IOPI Medical LCC, Redmond, WA, USA) was used to measure the lingual and labial strength. The IOPI is characterized by a high test-retest reliability and is frequently utilized in healthy adults and patients with various diagnoses [4,23]. This method also exhibits a high inter- and intra-rater reliability [9,24,25].

The examiner demonstrated the lingual and labial strength task and permitted the participant to squeeze a tongue bulb with his or her fingers prior to the participant performing the task. This intermediate step was important to facilitate familiarity and cooperation. Each participant was allowed to practice twice prior to any data collection. A new bulb was used for every participant because of hygienic concerns and to minimize measurement errors due to possible compliance variations in the bulb after extended use.

### 2.3. Procedures

Table 1 shows the eleven measuring procedures (for the three parts) developed to assess the MIPs, tongue endurance, and swallowing pressure [20,23,26]. All of the labial and lingual strength parameters were measured 3 times. The maximal value of three trials represents the pressure of every parameter. Participants who complained of orofacial musculature soreness during the assessment stopped performing the measurement and were excluded.

#### 2.3.1. Maximum Isometric Pressures

The evaluation of the lingual pressure included the MIP of the anterior and posterior tongue. The tongue bulb placement was located behind the central incisors (anterior tongue) and aligned with the first molars (posterior tongue) [26]. The examiner placed a permanent mark directly on the participant’s closed lips on the plastic tubing attached to the tongue bulb in both the anterior and posterior lingual regions. As such, the examiner could monitor the tongue bulb placement for consistency across all trials [23,27].

Tongue strength, or MIP, was measured as the greatest pressure exerted across three consecutive trials at both the anterior and posterior lingual regions. The participants were requested to squeeze the bulb between the tongue and hard palate with maximum effort for approximately 2 s. The participants were allowed to rest for 10 s between trials.

Labial strength was measured by placing the tongue bulb inside the cheek immediately lateral to the corner of the mouth, and the participants squeezed the bulb against the buccal surface of the teeth by pouting the lips as hard as possible [28].

#### 2.3.2. Tongue Endurance

Following the completion of the tongue and labial strength measurements, the participants were allowed to rest for 1 min. Tongue endurance was the period of time when the participants maintained 50% of their MIP for the anterior and posterior tongue. For tongue endurance, the LCD screen on the IOPI displayed the exerted pressure in kPa and the LED lights displayed how long the participants held the top (green) light on to signify tongue endurance.

#### 2.3.3. Swallowing Pressure

Swallowing pressure was defined as the non-effortful swallowing pressure across three consecutive trials for saliva swallowing and thin liquid (commercially bottled water) swallowing. The bolus was offered by the investigator in a cup (5 mL) and swallowed by the participant with the tongue bulb in the labial and lingual regions.

### 2.4. Statistical Analysis

All the descriptive and inferential statistical analyses were performed using SPSS version 23.0 (IBM Corp., Armonk, NY, USA). The significance level was set at 0.05. The normality of the data was investigated using a histogram, normal quantile–quantile (Q–Q) plots, skewness, kurtosis, and the Shapiro–Wilk test [29]. Descriptive statistics (means and 95% confidence intervals (C)] of the mean, and minimum and maximum values) were calculated for all the variables. A repeated-measures analysis of variance (ANOVA) was performed to evaluate the effect of three trials in attaining the labial and lingual strength.

## 3. Results

Of the 150 recruited participants, 49 were males and 101 were females (age range: 20–79 years; mean ± standard deviation (SD): 36.1 ± 14.9 years). There were no participants with oral motor disease. More than half of the participants (54.0%) had an abnormal body mass index value (<18.5 or >24.0 kg/m^2^) (Table 2). No participant was excluded for orofacial musculature soreness during measurement.

### 3.1. The Normality Test of Variables

**Figure 1:**
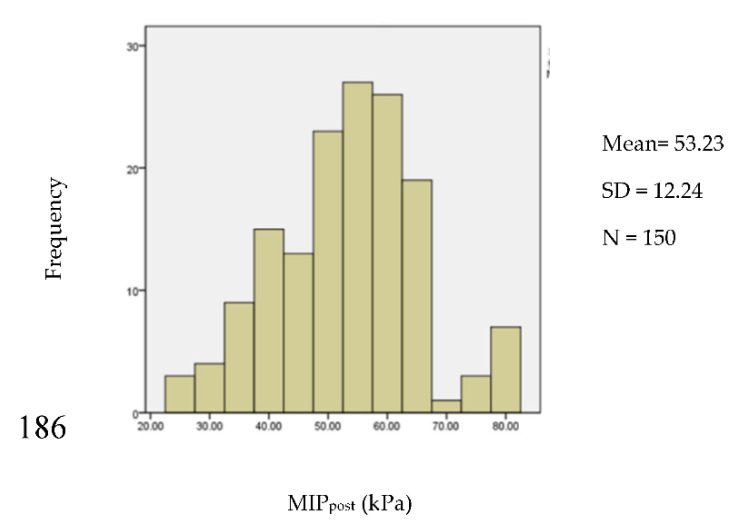
The histogram of the maximum isometric pressure of the posterior tongue.

**Figure A1:**
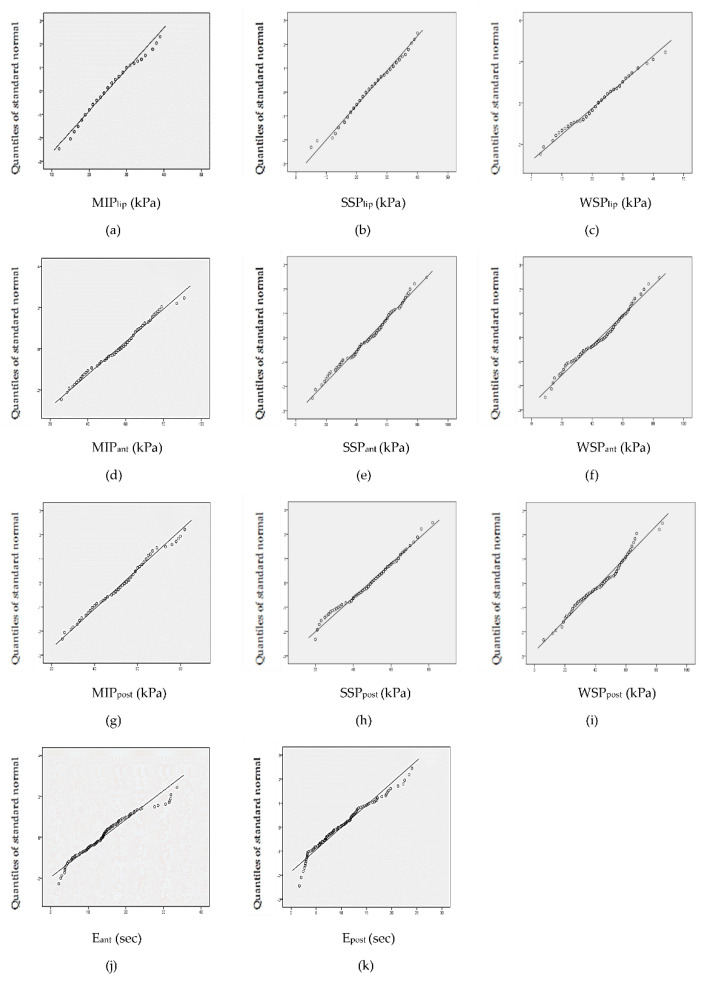
Quantile-quantile (Q-Q) plots of the dependent variables. The 95% confidence intervals are indicated by the dotted lines. (**a**) Q-Q plot of MIP_lip_; (**b**) Q-Q plot of SSP_lip_; (**c**) Q-Q plot of WSP_lip_; (**d**) Q-Q plot of MIP_ant_; (**e**) Q-Q plot of SSP_ant_; (**f**) Q-Q plot of WSP_ant_; (**g**) Q-Q plot of MIP_post_; (**h**) Q-Q plot of SSP_post_; (**i**) Q-Q plot of WSP_post_; (**j**) Q-Q plot of E_ant_; (**k**) Q-Q plot of E_post_.

There were several different methods used to test the normality of data on the maximum isometric pressures and swallowing pressures of both the lips and tongue, and tongue endurance, including the analysis of the histogram, Q–Q plots, skewness, kurtosis, and Shapiro–Wilk test (Table 3). For example, the histogram for the MIP of the posterior tongue (MIP_post_) indicated symmetry (*p* = 0.079), and the spread of the sample illustrated a normal distribution (Figure 1). The Q–Q plot (Figure A1g in Appendix A) compared the ordered distribution of the MIP_post_ with the quantiles of a standard normal distribution indicated by the straight line. Figure A1g shows a normal distribution because the points lie along the line.

Data analysis revealed some outliers in the measurement of labial strength and tongue endurance. Outliers in statistical analyses are extreme values that do not appear to fit with the majority of a data set. If not removed, these extreme values can have a large effect on any conclusions that may be drawn from the data in question, because they can skew the correlation coefficients and lines of best fit in the wrong direction. After removing the outliers, nonsignificant Shapiro–Wilk tests were observed in the data for the SSP of the lips (SSP_lip_) (n = 145) (*p* = 0.190) and water swallowing pressure (WSP) of the lips (WSP_lip_) (n = 144) (*p* = 0.197) (Table 3). A normal distribution was not uniformly present even after removing the outliers for the measurements of the MIP of the lips (MIP_lip_) (n = 145), WSP of the anterior tongue (WSP_ant_) (n = 150) or posterior tongue (WSP_post_) (n = 150), and endurance of the anterior tongue (E_ant_) (n = 132) or posterior tongue (E_post_) (n = 136) (Table 3).

There were no outliers removed in the measurements of tongue strength for both anterior and posterior locations. The datasets for the MIP of the anterior (MIP_ant_) (*p* = 0.127) and posterior (MIP_post_) (*p* = 0.079) tongue strength were normally distributed. It is common to consider values below the 5th percentile to be “abnormal” [22,30]. The formula (mean − 1.65 × SD) [31] can subsequently be used to determine the cut-off point from these data (e.g., 55.95 − 1.65 × 14.13 = 32.64 for anterior tongue strength; and 53.23 − 1.65 × 12.24 = 33.0 for posterior tongue strength). Values <33 kPa indicate a weak tongue strength (Table 4).

### 3.2. Labial and Lingual Strength Pattern

Descriptive statistics (mean, SD, and 95% CI) for the generation of labial and lingual pressure in the total sample are reported in Table 3. The average MIP_lip_, MIP_ant_, and MIP_post_ were 24.81 ± 5.64, 55.95 ± 14.13, and 53.23 ± 12.24 kPa, respectively. The mean value of posterior tongue strength was less than that of the anterior tongue strength by approximately 5%. The average SSP_lip_, SSP_ant_, and SSP_post_ were 23.82 ± 6.91, 47.91 ± 15.29, and 48.11 ± 14.91 kPa, respectively. The average WSP_lip_, WSP_ant_, and WSP_post_ were 21.95 ± 7.89, 45.16 ± 16.21, and 43.38 ± 15.42 kPa, respectively. The average E_ant_ and E_post_ were 13.86 ± 7.08 and 10.06 ± 5.40 s, respectively.

The average swallowing pressures were divided by the MIP for each bolus and multiplied by 100 to derive the proportion or percentage of the maximum isometric lip or tongue pressure generated during the swallowing trials. Hence, the percentages of the maximum isometric lip pressure generated during the swallowing of saliva and water were 96% and 88%, respectively. The percentages of the maximum isometric tongue pressure generated during the swallowing of saliva and water were 85% and 80% for the anterior tongue, and 90% and 81% for the posterior tongue, respectively.

### 3.3. Repeated Trials of Labial and Lingual Strength

A repeated-measures analysis of covariance was used to analyze the differences among three trials of labial and lingual strength parameters. Prior to performing repeated measurements of ANOVAs, the values obtained from the three trials were subject to spherical verification; *p* > 0.05 denotes that the three repeated measurements are not correlated and conform to spherical verification. In contrast, *p* < 0.05 denotes that the three measurements are highly correlated and do not conform to spherical verification; hence, they must be corrected using the Greenhouse–Geisser method. When repeated measurements of ANOVAs showed significant differences, the least significant difference was further used to perform pairwise comparisons among the three measurement values. These results did not show a statistically significant difference for any of the pressures obtained from the labial and lingual strength parameters (Table 5).

## 4. Discussion

This study aimed to investigate and establish reference values for the labial and lingual strength of healthy Chinese adults. It is important to point out that the values for tongue strength and endurance in the present study fell within the normative range of 40–80 kPa and >10 s, respectively [5,13,20]. The mean MIP_ant_ in this Chinese population was 55.95 kPa (CI: 53.67–58.22). In a Belgian population, Vanderwegen et al. found a mean of 44.27 kPa (CI: 42.83–45.71) [20]. In an American population, Stierwalt and Youmans reported a mean of 59.78 kPa (CI: 57.88–61.68) [13]. The average MIP_post_ of 53.23 kPa (CI: 51.26–55.21) observed in our study was also higher than that reported by Vanderwegen et al. (mean: 41.08 kPa; CI: 39.67–42.48) [20], but similar to that found by Clark and Solomon (mean: 53.60 kPa; CI: 51.28–55.92) [5]. However, compared with this study, the overall measurements of maximal tongue strength in a Korean population were lower by approximately 10 kPa [32]; the mean E_ant_ was 13.86 s (CI: 12.64–15.07), which is lower than that recorded by Vanderwegen et al. (mean: 22.39 s; CI: 20.78–24.12) [20]. The mean E_post_ in our study group was 10.06 s (CI: 9.14–10.97), while Vanderwegen et al. [20] observed a mean of 14.90 s (CI: 13.96–15.90). In contrast, the overall measurements of tongue endurance across different age decades in the Korean population were found to be higher than those revealed by our study [32].

Maximum tongue strength declines with advanced age and is lower in females [17,18]. The average age of participants in our study and in the studies conducted by Stierwalt and Youmans [13], Clark and Solomon [5], and Vanderwegen et al. [20] was 36.1 ± 14.9, 42.34 ± 20.3, 43.79 ± 20.4, and 54.8 ± 20.9 years, respectively. This may explain why the value of the tongue strength measurement in the study conducted by Vanderwegen et al. [20] was the lowest. Although the average age of participants in the current study was the lowest one, the tongue endurance value was the lowest one. In terms of gender ratio, the male to female ratio of the current study and the studies conducted by Stierwalt and Youmans [13], Clark and Solomon [5], Vanderwegen et al. [20], and Jeong et al. [32] was 49:101, 80:20, 88:83, 210:210, and 60:60, respectively. Data on the effects of age and gender on tongue endurance are relatively scarce, and most often indicate no effects with advanced age and gender [13,20,32]. The present study is a comparative research on labial and lingual strength among Chinese, European, American, and Korean populations. This issue merits further research in different regional standard values and cross-national surveys of tongue strength with considering diverse perspectives, including age, gender, race, and nationality.

In the present study, the value of 33 kPa was used to demarcate a weak tongue strength for this Chinese population; this value is lower than those reported in previous studies [4,9,13]. The literature suggests that healthy adults should have maximum isometric tongue pressures ≥ 40 kPa, and SSPs between 20 and 30 kPa [13]. The percentages of maximum isometric tongue pressure generated during saliva swallowing and water swallowing were 40–60% [9,33]. However, Peladeau-Pigeon et al. analyzed the swallowing tasks of 84 healthy participants and found that the peak amplitudes of saliva swallowing, on average, ranged from 70% to 81% of the values obtained for the MIP_ant_ [34]. These findings were more similar to those of the present study (i.e., using > 80% of the total tongue MIP range during saliva and water swallowing). Although the participants were guided to perform saliva and water swallowing as naturally as possible, we cannot preclude the possibility that the prior performance of lingual maximum isometric tasks influenced the use of effort during the saliva and water swallowing tasks.

Future studies with the aim to shed light on the influence of racial differences on normal oral swallowing are warranted. Although maximal tongue strength decreases gradually with aging, tongue strength during swallowing remains static. It may impact on the swallowing pressure reserve. Hence, it is strongly suggested that the Chinese population should perform tongue exercises early to improve the maximum isometric tongue pressure and swallowing pressure reserve. The maximum isometric contraction of tongue and the activity of pressing the palate during the oral phase of saliva and water swallowing help in the comprehension of swallowing physiology, and will thus contribute to therapeutic planning for individuals with dysphagia. Owing to its convenience and limited invasiveness, maximum isometric tongue pressure may be useful for the early detection of presbyphagia in the general public.

In this study, there were 11 muscle measuring procedures, each of which was evaluated for three consecutive trials as recommended in the IOPI user manual, and a total 33 trials for muscle strength measurements. Following the completion of measurements, some participants experienced orofacial muscle soreness for 24 h. Similar results were reported in previous studies with tongue strength measurements [21,22]. Repeated trials had no effect on the measurement of labial and lingual strength parameters; this result was in line with the findings reported by Vanderwegen et al. [20]. Previous research also demonstrated that the correlation between mean pressure and maximal pressure across the three trials of tongue strength is strong [25]. Since both are related to the oral phase swallowing function, the use of the maximal pressure of the three trials is more efficient in a clinical setting because there is no need for calculation.

There was no significant difference in the three trials in terms of the anterior and posterior tongue strength and labial strength for the MIP, saliva swallowing, water swallowing, and endurance of the tongue. Owing to the variability of the participants, the SDs of the measurement values ranged widely (7.16–21.46 kPa) (Table 5). The measurement of tongue strength and labial strength will be popularized to clinical practice in the future for patients, individuals with physical disability, or older adults. The reliability and validity of the measured values are essential; therefore, it is advisable to have a conservative attitude. In addition to specifying the location of the bulb and the procedure prior to measurement, the participants should practice placing the pressure bulb in the mouth and become familiar with the feeling of the pressure bulb in the mouth. Once the participants have the opportunity to practice before the formal measurement, the formal measurement can be performed once or twice to reduce participant fatigue.

The present study had several limitations that should be acknowledged. This sample was composed of healthy adults recruited from the general public. Therefore, this dataset may have been vulnerable to volunteer bias. The number of older adults was only 10, and the data were insufficient to assume the statistical normality of the distribution regarding tongue and lip strength in the normal population. In addition, the female percentage (101) was two times higher than the male (49), and this could have affected the results. Future research should recruit participants who are evenly distributed among different age and gender categories. Thus, the data can be broken down by age group and sex to help elucidate the findings and compare with previous publications.

## 5. Conclusions

This study explored the reference values of lingual and labial strength measurements in different tasks for healthy Chinese adults. The maximum isometric pressures of the lips, anterior tongue, and posterior tongue were 24.81 ± 5.64, 55.95 ± 14.13, and 53.23 ± 12.24 kPa, respectively. The percentages of maximum isometric tongue pressure during the swallowing of saliva and water were 85% and 80% for the anterior tongue and 90% and 81% for the posterior tongue, respectively. The average endurances for the anterior tongue and posterior tongue were 13.86 ± 7.08 and 10.06 ± 5.40 s, respectively. As for the repeated trials of labial and lingual strength, there were no statistically significant differences for any of the pressures obtained from the 11 labial and lingual strength parameters.

Large samples of data that quantify a range of normal tongue function are important to serve as a basis of comparison for individuals with impairments. Therefore, a larger sample size is necessary to extend the findings of this study to the general healthy population in China. Extensive population examination including abled and disabled participants would be recommended for establishing normative data.

## Figures and Tables

**Table 1:** Measuring procedures for the maximum isometric pressure, tongue endurance, and swallowing pressure.

Part	Item	Bulb Placement	Measuring Procedure
Part 1: Maximum isometric pressure	1. Maximum isometric pressure of the lips.	Tongue bulb placed inside the cheek, immediately lateral to the corner of the mouth.	Participants squeezed the bulb against the buccal surface of the teeth by pouting the lips as hard as possible.
	2. Maximum isometric pressure of the anterior tongue.	Tongue bulb placed behind the central incisors.	1. Participants were requested to squeeze the bulb between the tongue and hard palate with maximum effort for nearly 2 s. 2. Participants were allowed to rest for 10 s between trials.
	3. Maximum isometric pressure of the posterior tongue.	Tongue bulb aligned with the first molars.	Participants were asked to follow the same measuring procedures used in the strength measurement of the anterior tongue.
Part 2: Tongue endurance	4. Endurance of the anterior tongue.	Same placement as used in the strength measurements.	1. The target force was 50% of the subject’s maximum isometric pressure of the anterior tongue. 2. Participants were required to press the bulb against the hard palate with the tongue as hard as necessary to sustain the target force for as long as possible. 3. Measurement was initiated when the pressure reached or exceeded the target force and terminated when the pressure dropped steeply < 50% of the target force for ≥0.5 s.
	5. Endurance of the posterior tongue.	Same placement as used in the strength measurements.	Participants were asked to follow the same measuring procedure used in the endurance measurement of the anterior tongue.
Part 3: Swallowing pressure	6. Saliva/water swallowing pressure of the lips.	Same placement as used in the strength measurements.	1. Place the bulb in the specified position. 2. The participants were asked to swallow their saliva or 5 mL of water in a comfortable manner.
	7. Saliva/water swallowing pressure of the anterior tongue.	Same placement as used in the strength measurements.	Participants were asked to follow the same measuring procedure used in the saliva/water swallowing pressure measurement of the lips.
	8. Saliva/water swallowing pressure of the posterior tongue.	Same placement as used in the strength measurements.	Participants were asked to follow the same measuring procedure used in the saliva/water swallowing pressure measurement of the lips.

Source: (1) References [20,23,26,28]. (2) Compiled by the authors.

**Table 2:** Characteristics of the participants (N = 150).

Variable	No (%)	Mean (SD)
Gender		
Male	49 (32.7)	
Female	101 (67.3)	
Age (years)		36.1 (14.9), Range: 20–79
20–29	67 (44.7)	
30–39	23 (16.7)	
40–49	30 (20.0)	
50–59	18 (12.0)	
Above 60	10 (6.7)	
Body mass index (BMI)		24.1 (3.9)
Normal (18.5–24.0)	69 (46.0)	
Abnormal	81 (54.0)	
Below 18.5	8 (5.4)	
Above 24.0	73 (48.6)	

**Table 3:** The normality test of variables without outliers.

Variables	Mean (SD)	95% CI Mean	Min	Max	Skewness	Kurtosis	Shapiro-Wilk *p ^a^*
Lip (kPa)							
MIP_lip_	24.81(5.64)	23.88–25.73	12	39	0.45	−0.14	0.012
SSP_lip_	23.82 (6.91)	22.68–24.94	5	40	0.05	−0.05	0.190
WSP_lip_	21.95 (7.89)	20.65–23.25	3	44	−0.08	0.02	0.197
Tongue (kPa)							
MIP_ant_	55.95 (14.13)	53.67–58.22	17	93	−0.05	0.37	0.127
SSP_ant_	47.91 (15.29)	45.43–50.37	11	86	−0.23	−0.38	0.130
WSP_ant_	45.16 (16.21)	42.54–47.77	9	84	−0.23	−0.69	0.014
MIP_post_	53.23 (12.24)	51.26–55.21	25	82	−0.01	−0.09	0.079
SSP_post_	48.11 (14.91)	45.70–50.51	8	82	−0.32	−0.31	0.089
WSP_post_	43.38 (15.42)	40.89–45.86	6	84	−0.16	−0.56	0.008
Endurance (sec)							
E_ant_	13.86 (7.08)	12.64–15.07	2.16	33.62	0.69	0.49	<0.001
E_post_	10.06 (5.40)	9.14–10.97	1.65	24.05	0.53	−0.31	<0.001

Note: MIP_lip_ = Maximum isometric pressure of lip (N = 145); MIP_ant_ = Maximum isometric pressure of anterior tongue (N = 150); MIP_post_ = Maximum isometric pressure of posterior tongue (N = 150); SSP_lip_ = Saliva swallowing pressure of lip (N = 145); SSP_ant_ = Saliva swallowing pressure of anterior tongue (N = 150); SSP_post_ = Saliva swallowing pressure of posterior tongue (N = 150); WSP_lip_ = Water swallowing pressure of lip (N = 144); WSP_ant_ = Water swallowing pressure of anterior tongue (N = 150); WSP_post_ = Water swallowing pressure of posterior tongue (N = 150); E_ant_= Endurance of anterior tongue (N = 132); E_post_ = Endurance of posterior tongue (N = 136); *p ^a^* > 0.05 means that the data are in a normal distribution.

**Table 4:** Normal values of tongue strength (kPa).

Region	1%	5%	10%	50%
Anterior tongue	23	33	38	55.95 (14.13)
Posterior Tongue	25	33	38	53.23 (12.24)

Note: (1) Anterior tongue strength, 5% one-tail value = mean – 1.65 × standard deviation = 55.95 − 1.65×14.13 = 32.64; (2) Posterior tongue strength, 5% one-tail value = mean – 1.65 × standard deviation = 53.23 − 1.65×12.24 = 33.0.

**Table 5:** Repeated trials for the labial and lingual pressure analysis.

Variable	M(SD)	Sphericity (*p*)	F	*p*
Lip (kPa)				
MIP_lip_		<0.001	F (1.69, 8.37) = 0.51	0.599
1st	23.31 (7.16)			
2nd	23.66 (7.33)			
3rd	23.71 (7.80)			
SSP_lip_		<0.001	F (1.72, 24.06) = 1.47	0.233
1st	22.82 (9.04)			
2nd	22.12 (8.30)			
3rd	22.26 (8.27)			
WSP_lip_		0.367	F (2, 13.54) = 1.09	0.338
1st	20.85 (9.34)			
2nd	20.82 (9.70)			
3rd	20.31 (8.95)			
Tongue (kPa)				
MIP_ant_		<0.001	F (1.81, 16.96) = 0.38	0.661
1st	51.11 (15.33)			
2nd	51.71 (14.70)			
3rd	51.59 (14.88)			
SSP_ant_		0.645	F (2, 49.02) = 1.02	0.360
1st	42.94 (16.81)			
2nd	41.94 (16.68)			
3rd	42.92 (15.40)			
WSP_ant_		0.003	F (1.86, 138.12) = 2.60	0.080
1st	38.89 (16.26)			
2nd	39.36 (16.01)			
3rd	40.67 (17.58)			
MIP_post_		<0.001	F (1.74, 15.51) = 0.44	0.619
1st	49.45 (12.38)			
2nd	49.32 (12.40)			
3rd	48.88 (13.37)			
SSP_post_		0.469	F (2, 26.13) = 0.71	0.494
1st	43.91 (15.72)			
2nd	43.45 (15.53)			
3rd	43.07 (14.81)			
WSP_post_		0.348	F (2, 0.21) = 0.01	0.994
1st	38.46 (16.22)			
2nd	38.51 (14.70)			
3rd	38.53 (15.45)			
Endurance (sec)				
E_ant_		<0.001	F (1.74, 290.86) = 1.24	0.288
1st	16.28 (21.46)			
2nd	14.45 (16.57)			
3rd	13.77 (13.84)			
E_post_		0.139	F (2, 136.76) = 1.07	0.345
1st	11.77 (18.53)			
2nd	11.04 (19.60)			
3rd	9.88 (10.54)
